# Aging of reward dopamine tracts in the human brain: A diffusion tensor imaging study

**DOI:** 10.1097/MD.0000000000036112

**Published:** 2023-11-17

**Authors:** Jeong Pyo Seo, Heun Jae Ryu

**Affiliations:** a Department of Physical Therapy, College of Health and Welfare Sciences, Dankook University, Cheonan, Republic of Korea; b Department of Public Health Sciences, Graduate School, Dankook University, Cheonan, Republic of Korea.

**Keywords:** aging, diffusion tensor imaging, diffusion tensor tractography, mesocortical tract, mesolimbic tract, reward tracts

## Abstract

The mesocortical tract (MCT) and mesolimbic tract (MLT) are reward dopaminergic tracts that have been shown to play a role in regulating reward stimuli, including both incentive salience and social stimuli. In the current study, we examined aging of the MCT and MLT in normal human participants to explain human brain structures using diffusion tensor tractography (DTT). Sixty-four healthy participants were recruited for this study and allocated to 3 groups based on participants’ age. Diffusion tensor imaging was performed, and MCTs and MLTs were reconstructed using the probabilistic tractography method. A significant negative correlation was observed between age and fractional anisotropy and tract volume of the MCT and MLT, whereas a positive correlation was observed between age and mean diffusivity. The mean fractional anisotropy value of the MCT was significantly lower in the old group than in the young and middle-aged groups (*P* < .05). The mean diffusivity values of the MCT and MLT were significantly higher in the old group than in the young and middle-aged groups (*P* < .05). The mean tract volume values of the MCT and MLT were significantly lower in the old group than in the young group (*P* < .05). We found that degenerative changes in the MCT and MLT began in participants in the 20s–30s, progressed steadily throughout life, and accelerated in the 60s.

## 1. Introduction

The mesocortical tract (MCT) and mesolimbic tract (MLT) are the major dopamine tracts involved in reward in the human brain.^[1]^ The MCT originates from the ventral tegmental area (VTA) and extends to the prefrontal cortex (PFC), including the orbitofrontal cortex, anterior cingulate cortex, and striatum. The MLT originates from the VTA and extends to the nucleus accumbens.^[1]^ These tracts are specifically important for rewards associated with cognitive control, motivation, and emotional responses.^[2]^

Previous research has highlighted the severe consequences of impairments in these tracts. For instance, anomalies in the MCT have been linked with increased white matter hyperintensities in drug addicts, affecting their decision-making and response inhibition capacities.^[3]^ Disruption of this pathway has also been documented extensively.^[4–8]^ The MLT, when compromised, leads to behavioral control issues, fostering cravings, withdrawal symptoms, and tolerance. Such impairments can result in nondrug addictions like gambling, shopping, and video game addictions.^[9]^ Additionally, it has been observed that MLT impairments can influence social interactions, leading to deficits in normal children compared to those with autism.^[10]^

Understanding the typical aging patterns of these specific brain structures is paramount. Detailed knowledge regarding normal aging of specific brain structures aids in the development of strategies aimed at preventing or delaying aging.^[11–14]^ Recently, many diffusion tensor tractography (DTT) studies have been performed because this modern technique enables three-dimensional qualitative visualization and analysis of neural structures. Age-related studies using DTT have also been performed.^[11–13]^ However, few such studies have focused on the MCT and MLT.^[15–23]^ To the best of our knowledge, no study has addressed the effects of aging on these tracts.

In the current study, we investigated age-related changes in the MCT and MLT among dopaminergic pathways using DTT to compare differences in aging as a neuroimaging technique in this study.

## 2. Methods

### 2.1. Participants

Sixty-four right-handed, healthy participants (males: 40, females: 24, mean age: 47.45 ± 17.69 years; range: 20–79 years) who had no previous history of neurological, psychiatric, or physical illness and no brain lesions on conventional MRI (T1-weighted, T2-weighted, fluid-attenuated inversion recovery, or T2-weighted gradient recall echo images), as confirmed by a neuroradiologist, were enrolled in the present study. Participants were divided into 3 groups based on participants’ age (young, middle-aged, and old). The young group included participants in their 20s and 30s, the middle-aged group included those in their 40s and 50s, and the old group included hose in their 60s and 70s.^[24]^ All participants provided written informed consent prior to study commencement, and the study protocol was approved by the institutional review board of the University Hospital.

### 2.2. DTI acquisition

Diffusion tensor imaging (DTI) data were acquired using a Synergy-L SENSE head coil on a 1.5T Gyroscan Intera system (Philips, Best, The Netherlands) equipped with single-shot echo-planar imaging. For each of the 32 non-collinear diffusion-sensitizing gradients, 67 contiguous slices were acquired parallel to the anterior commissure–posterior commissure line. The imaging parameters were as follows: acquisition matrix = 96 × 96, reconstructed matrix = 192 × 192 matrix, field of view = 240 × 240 mm^2^, TR = 10,398 ms, TE = 72 ms, parallel imaging reduction factor (SENSE factor) = 2, EPI factor = 59 and b = 1000 s/mm^2^, NEX = 1, slice gap = 0, and slice thickness 2.5 mm.

### 2.3. Fiber tracking

The Oxford Centre for functional magnetic resonance imaging of the brain software library (FSL; www.fmrib.ox.ac.uk/fsl) was used to analyze the diffusion-weighted imaging data.^[25,26]^ Affine multi-scale two-dimensional registration was used to correct for head motion effects and image distortions caused by eddy currents. A probabilistic tractography method based on a multi-fiber model was used for fiber tracking and was applied utilizing tractography routines implemented in functional magnetic resonance imaging of the brain’s Diffusion Toolbox (step length 0.5 mm, 5000 streamline samples, curvature threshold = 0.2).^[26–29]^

**Figure 1.**
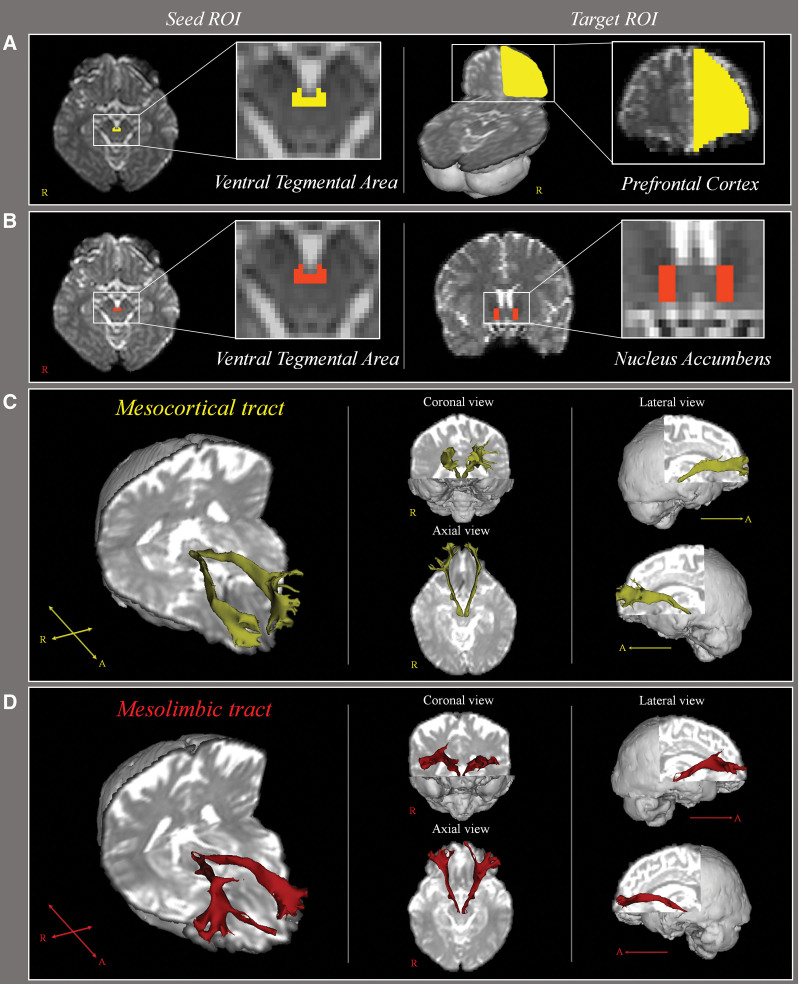
(A) Seed regions of interest (ROIs) for mesocortical tract were placed on the ventral tegmental area (yellow); target ROIs for mesocortical tract were placed on the prefrontal cortexs (yellow); (B) seed regions of interest (ROIs) for mesolimbic tract were placed on the ventral tegmental area (red); target ROIs for mesolimbic tract were placed on the nucleus accumbens (red); (C) the reconstructed mesocortical tract; (D) the reconstructed mesolimbic tract.

The MCT and MLT were delineated by selecting fibers that passed through the seed and target regions of interest (ROIs). For each participant, as in the MCT, the seed ROI was placed on the VTA in the midbrain, and the target ROI was located in the PFC at the inferior frontal sulcus (Fig. 1).^[30]^ For the MLT, the seed ROI was placed on the VTA in the midbrain, and the target ROI was located in the nucleus accumbens of the VS (Fig. 1).^[30]^ Fractional anisotropy (FA), mean diffusivity (MD), and tract volume (TV) of the MCT and MLT were measured.

### 2.4. Statistical analysis

Data were analyzed using SPSS software (version 25.0; SPSS Inc., Chicago, IL). Pearson correlation analysis was performed to assess the significance of the correlations between the 3 DTI parameters (FA, MD, and TV) and age. Statistical significance was set at *P* < .05. The Kolmogorov–Smirnov test was used to test for normality among the outcome measures. One-way ANOVA with Bonferroni post hoc test was used to determine the significance of differences for each DTI parameter (FA, MD, and TV) between the 3 groups. Statistical significance was set at *P* < .05.

## 3. Results

**Figure 2.**
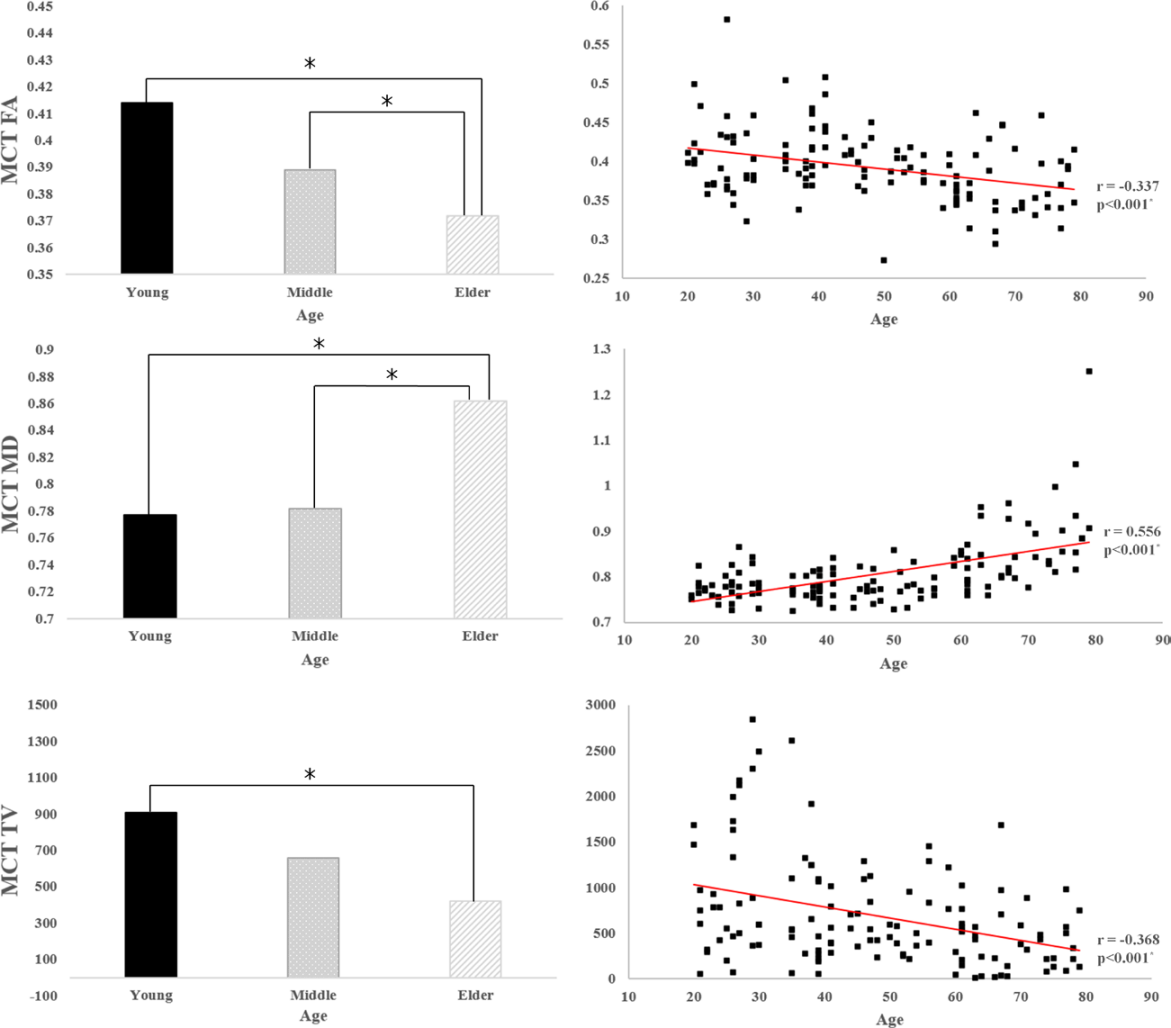
(Left) Results of Bonferroni post hoc test in terms of the mesocortical tract among age groups; (right) correlations between age and the 3 DTT parameters of mesocortical tract. DTT = diffusion tensor tractography.

The correlations between age and the DTI parameters of the MCT are shown in Figure 2. We observed a significant moderate positive correlation between age and MD of the MCT (*R* = 0.556, *P* < .05) and a significantly weak negative correlation between age and FA and TV values of the MCT (r = −0.337, r = −0.368, *P* < .05) (Fig. 2). In addition, there were significant differences in the mean FA, MD, and TV values of the MCT among the 3 groups (*P* < .05) (Fig. 2) (Table 1). The mean FA values of the MCT were higher in the young and middle-aged groups than in the old group (*P* < .05). The mean MD values of the MCT were lower in the young and middle-aged groups than in the old group (*P* < .05). TV values of the MCT were higher in the young group than in the old group (*P* < .05).

**Table 1:** Comparison of diffusion tensor image parameters of the mesocortical tract among the 3-age group.

Group	FA	MD	TV
Young	0.414 (0.038)	0.777 (0.029)	909.269 (731.787)
Middle	0.389 (0.050)	0.782 (0.035)	656.353 (347.371)
Elder	0.372 (0.041)	0.862 (0.902)	419.714 (349.677)
*P*-value	.001*	<.001*	<.001*
Post hoc	Y, M > E	Y, M < E	Y > E

Values indicate mean (±standard deviation).

E = elder aged group, FA = fractional anisotropy, M = middle aged group, MD = mean diffusivity (MD × 10^−3^ [mm^2^/s]), TV = tract volume, Y = young aged group.

* *P* < .05.

**Figure 3.**
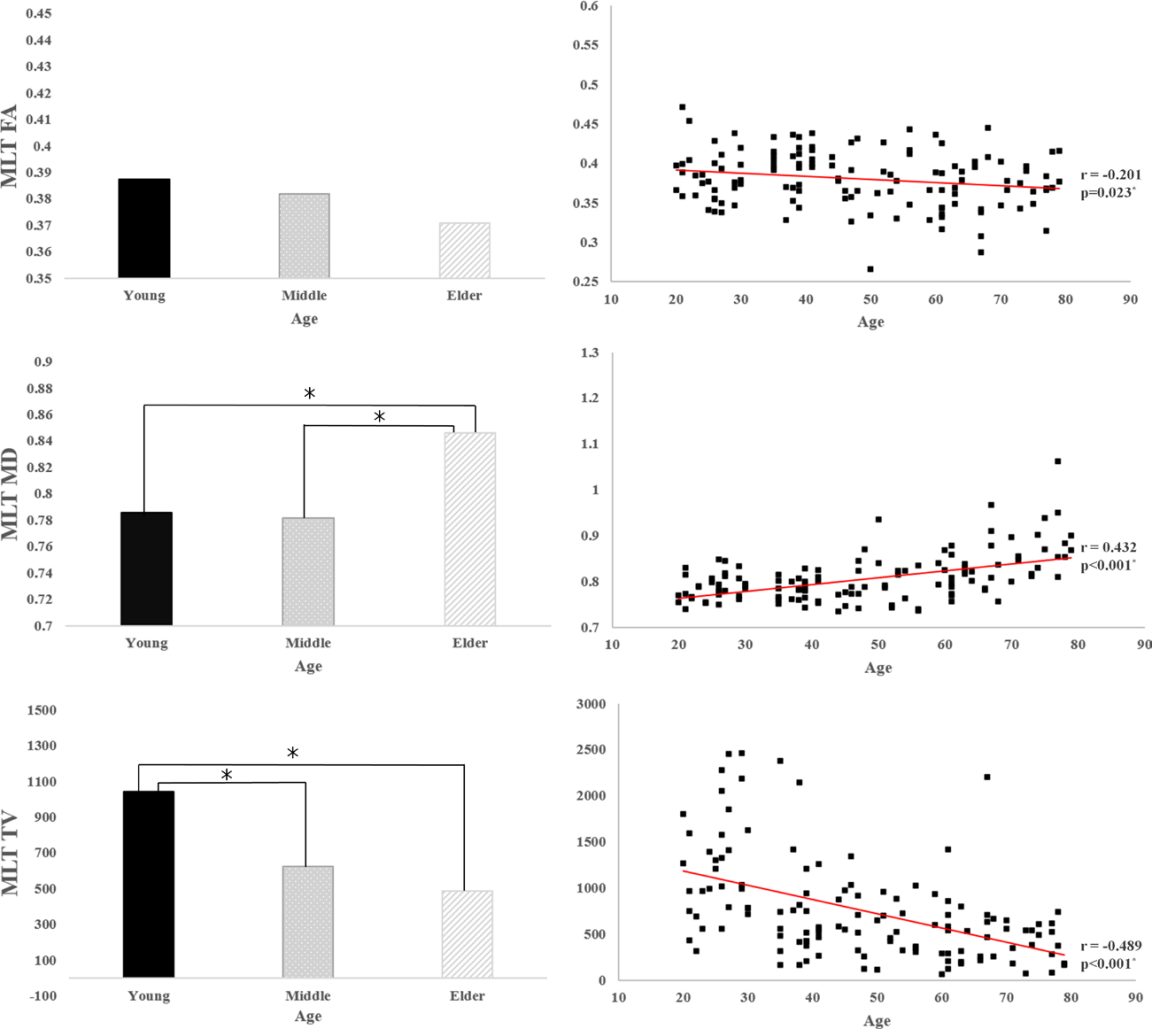
(Left) Results of Bonferroni post hoc test in terms of the mesolimbic tract among age groups; (right) correlations between age and the 3 DTT parameters of mesolimbic tract. DTT = diffusion tensor tractography.

In terms of the MLT, a significant moderate positive correlation was observed between age and MD of the MLT (*R* = 0.432, *P* < .05) and a significant weak and moderate negative correlation between age and FA and TV values of the MLT (r = −0.201, r = −0.489, *P* < .05) (Fig. 3). Among the 3 groups, there were significant differences in both MD and TV values of the MLT (*P* < .05) (Fig. 3) (Table 2). The mean MD values of the MLT were lower in the young and middle-aged groups than in the old group (*P* < .05). TV values of the MLT were higher in the young group than in the other groups (*P* < .05). However, the mean FA values of the MLT were not significantly different among the 3 groups (*P* > .05).

**Table 2:** Comparison of diffusion tensor image parameters of the mesolimbic tract among the 3-age group.

Group	FA	MD	TV
Young	0.387 (0.031)	0.786 (0.026)	1043.058 (621.677)
Middle	0.382 (0.039)	0.782 (0.074)	624.029 (309.107)
Elder	0.371 (0.035)	0.846 (0.061)	486.405 (379.542)
*P*-value	.066	<.001*	<.001*
Post hoc	–	Y, M < O	Y > M, O

Values indicate mean (±standard deviation)

E = elder aged group, FA = fractional anisotropy, M = middle aged group, MD = mean diffusivity (MD × 10^−3^ [mm^2^/s]), TV = tract volume, Y = young aged group.

* *P*<.05.

## 4. Discussion

We used DTI to compare differences in aging as a neuroimaging technique by investigating age-related changes in the MCT and MLT among dopaminergic pathways. We obtained values of 3 DTI parameters: FA, MD, and TV. FA values refer to the degree of directionality and integrity of white matter microstructures, such as axons, myelin, and microtubules.^[31,32]^ In contrast, the MD values reflect the magnitude of water diffusion.^[31,32]^ TV values indicate the total number of fibers in a neural pathway.^[31,33]^ In other words, reducing the FA and TV values and increasing the MD values could be considered “degeneration.”^[34,35]^

We found an overall decreasing tendency in the FA and TV and increasing tendency in the MD of the MCT and MLT with aging. In terms of the MCT, FA values of the old group were significantly lower than those of the young and middle-aged groups. According to TV values, the old group had significantly lower TV values than the young group. Moreover, MD values of the old group were significantly greater than those of the young and middle-aged groups. This suggests that the decline in TV of the MCT begins around the age of 20 years. Reduction in FA and increase in MD of the MCT indicated that demyelination of the MCT accelerated around the age of 60 years. In case of the MLT, FA values were not significantly different among the groups, but MD values in the old group were significantly higher than those in the other groups. TV values of the old group were significantly lower than those of the other groups. Therefore, the increase in MD of the MLT began around the age of 60 years, and the decline in TV of the MLT began around the age of 20 years. This indicates that demyelination of the MLT began around the age of 20 years and accelerates around the age of 60 years. Based on the findings of this study, it was assumed that MCT degeneration began in the 20s and accelerated in the 60s, while MLT degeneration began in the 20s and accelerated in the 60s.

The MCT and MLT are parts of dopaminergic circuits that promote reward-motivated learning; dopamine released from the midbrain affects encoding memory.^[36–42]^ Age-related changes affect dopaminergic manipulation;^[20,43]^ hence, aging dopaminergic circuits have a strong correlation with memory decline. In contrast, VTA neurons responsive to reward-associated stimuli that were activated during social interaction and dopaminergic neurons, such as the MCT and MLT related to the PFC, have been shown to have an important relationship with social play behavior.^[44,45]^ In addition, normal older adults exhibit a reduction in their social activity and interest,^[46]^ which may be caused by a decline in social stimuli rewards relevant to aging of the MCT and MLT. Therefore, this finding might be an extension of the results these prior studies by showing that the connectivity of the structural pathway of the MCT and MLT is not only debilitated in older adults, but is also related to aging differences in the role of dopaminergic tracts, as previously reported.

In this study, we investigated age-related changes in the MCT and MLT using DTT and found that degenerative changes in the MCT and MLT were accelerated in the 60s. However, this study had some limitations. First, the number of groups was not matched. Second, the small number of subjects recruited in each group limits the generalizability of the results. Third, it is difficult to determine a clinical correlation because there is no clinically relevant evidence. Therefore, further studies that match a large number of subjects in each group and correlate clinical data are needed to compensate for these limitations.

## Author contributions

**Conceptualization:** Jeong Pyo Seo.

**Data curation:** Jeong Pyo Seo.

**Formal analysis:** Heun Jae Ryu.

**Investigation:** Heun Jae Ryu.

**Visualization:** Heun Jae Ryu.

**Writing – original draft:** Heun Jae Ryu.

## References

[1] Ikemoto S Panksepp J. The role of nucleus accumbens dopamine in motivated behavior: a unifying interpretation with special reference to reward-seeking. Brain Res Brain Res Rev. 1999;31:6–41. 10611493 10.1016/s0165-0173(99)00023-5

[2] Nestler EJ Hyman SE Malenka RC. Molecular Neuropharmacology: A Foundation for Clinical Neuroscience. New York: McGraw-Hill Medical. 2009; 147–148,367,376.

[3] Kaufman JN Ross TJ Stein EA. Cingulate hypoactivity in cocaine users during a GO-NOGO task as revealed by event-related functional magnetic resonance imaging. J Neurosci. 2003;23:7839–43. 12944513 10.1523/JNEUROSCI.23-21-07839.2003 PMC6740597

[4] Adinoff B Devous MD Sr Best SM. Limbic responsiveness to procaine in cocaine-addicted subjects. Am J Psychiatry. 2001;158:390–8. 11229979 10.1176/appi.ajp.158.3.390

[5] Bae SC Lyoo IK Sung YH. Increased white matter hyperintensities in male methamphetamine abusers. Drug Alcohol Depend. 2006;81:83–8. 16005161 10.1016/j.drugalcdep.2005.05.016

[6] Kim MJ Lyoo IK Kim SJ. Disrupted white matter tract integrity of anterior cingulate in trauma survivors. Neuroreport. 2005;16:1049–53. 15973146 10.1097/00001756-200507130-00004

[7] Volkow ND Fowler JS Wang G-J. Dopamine in drug abuse and addiction: results from imaging studies and treatment implications. Mol Psychiatry. 2004;9:557–69. 15098002 10.1038/sj.mp.4001507

[8] Volkow ND Wang GJ Ma Y. Expectation enhances the regional brain metabolic and the reinforcing effects of stimulants in cocaine abusers. J Neurosci. 2003;23:11461–8. 14673011 10.1523/JNEUROSCI.23-36-11461.2003 PMC6740524

[9] Trezza V Damsteegt R Achterberg EM. Nucleus accumbens μ-opioid receptors mediate social reward. J Neurosci Res. 2011;31:6362–70. 10.1523/JNEUROSCI.5492-10.2011 PMC3098965 21525276

[10] Supekar K Kochalka J Schaer M. Deficits in mesolimbic reward pathway underlie social interaction impairments in children with autism. Brain. 2018;141:2795–805. 30016410 10.1093/brain/awy191 PMC6113649

[11] Gunbey HP Ercan K Findikoglu AS. The limbic degradation of aging brain: a quantitative analysis with diffusion tensor imaging. Sci World J. 2014;2014:1–7. 10.1155/2014/196513 PMC4009154 24977184

[12] Stadlbauer A Salomonowitz E Strunk G. Quantitative diffusion tensor fiber tracking of age-related changes in the limbic system. Eur Radiol. 2008;18:130–7. 17701181 10.1007/s00330-007-0733-8

[13] Sullivan EV Pfefferbaum A. Diffusion tensor imaging and aging. Neurosci Biobehav Rev. 2006;30:749–61. 16887187 10.1016/j.neubiorev.2006.06.002

[14] Sullivan EV Rohlfing T Pfefferbaum A. Quantitative fiber tracking of lateral and interhemispheric white matter systems in normal aging: relations to timed performance. Neurobiol Aging. 2010;31:464–81. 18495300 10.1016/j.neurobiolaging.2008.04.007 PMC2815144

[15] Aalto S Brück A Laine M. Frontal and temporal dopamine release during working memory and attention tasks in healthy humans: a positron emission tomography study using the high-affinity dopamine D2 receptor ligand [11C] FLB 457. J Neurosci. 2005;25:2471–7. 15758155 10.1523/JNEUROSCI.2097-04.2005 PMC6725173

[16] Adcock RA Thangavel A Whitfield-Gabrieli S. Reward-motivated learning: mesolimbic activation precedes memory formation. Neuron. 2006;50:507–17. 16675403 10.1016/j.neuron.2006.03.036

[17] Drouin-Ouellet J Gibrat C Bousquet M. The role of the MYD88-dependent pathway in MPTP-induced brain dopaminergic degeneration. J Neuroinflamm. 2011;8:1–12. 10.1186/1742-2094-8-137 PMC3203853 21989292

[18] Knecht S Breitenstein C Bushuven S. Levodopa: faster and better word learning in normal humans. Ann Neurol. 2004;56:20–6. 15236398 10.1002/ana.20125

[19] Lisman JE Grace AA. The hippocampal-VTA loop: controlling the entry of information into long-term memory. Neuron. 2005;46:703–13. 15924857 10.1016/j.neuron.2005.05.002

[20] Petersen RC Smith GE Waring SC. Aging, memory, and mild cognitive impairment. Int Psychogeriatr. 1997;9:65–9. 10.1017/s1041610297004717 9447429

[21] Raghunathan R Polinski NK Klein JA. Glycomic and proteomic changes in aging brain nigrostriatal pathway. Mol Cell Proteomics. 2018;17:1778–87. 29915149 10.1074/mcp.RA118.000680 PMC6126385

[22] Schott BH Seidenbecher CI Fenker DB. The dopaminergic midbrain participates in human episodic memory formation: evidence from genetic imaging. J Neurosci. 2006;26:1407–17. 16452664 10.1523/JNEUROSCI.3463-05.2006 PMC6675495

[23] Seo JP Koo DK. Aging of the nigrostriatal tract in the human brain: a diffusion tensor imaging study. Medicina (Kaunas). 2021;57:994. 34577917 10.3390/medicina57090994 PMC8464776

[24] Ota M Obata T Akine Y. Age-related degeneration of corpus callosum measured with diffusion tensor imaging. Neuroimage. 2006;31:1445–52. 16563802 10.1016/j.neuroimage.2006.02.008

[25] Puig J Pedraza S Blasco G. Acute damage to the posterior limb of the internal capsule on diffusion tensor tractography as an early imaging predictor of motor outcome after stroke. AJNR Am J Neuroradiol. 2011;32:857–63. 21474629 10.3174/ajnr.A2400 PMC7965569

[26] Smith SM Jenkinson M Woolrich MW. Advances in functional and structural MR image analysis and implementation as FSL. Neuroimage. 2004;23:S208–19. 15501092 10.1016/j.neuroimage.2004.07.051

[27] Behrens TE Berg HJ Jbabdi S. Probabilistic diffusion tractography with multiple fibre orientations: what can we gain? Neuroimage. 2007;34:144–55. 17070705 10.1016/j.neuroimage.2006.09.018 PMC7116582

[28] Behrens TE Johansen-Berg H Woolrich M. Non-invasive mapping of connections between human thalamus and cortex using diffusion imaging. Nat Neurosci. 2003;6:750–757. 12808459 10.1038/nn1075

[29] Kunimatsu A Aoki S Masutani Y. The optimal trackability threshold of fractional anisotropy for diffusion tensor tractography of the corticospinal tract. Magn Reson Med Sci. 2004;3:11–7. 16093615 10.2463/mrms.3.11

[30] Nakamura-Palacios EM Lopes IBC Souza RA. Ventral medial prefrontal cortex (vmPFC) as a target of the dorsolateral prefrontal modulation by transcranial direct current stimulation (tDCS) in drug addiction. J Neural Transm (Vienna). 2016;123:1179–94. 27138429 10.1007/s00702-016-1559-9

[31] Assaf Y Pasternak O. Diffusion tensor imaging (DTI)-based white matter mapping in brain research: a review. J Mol Neurosci. 2008;34:51–61. 18157658 10.1007/s12031-007-0029-0

[32] Neil JJ. Diffusion imaging concepts for clinicians. J Magn Reson Imaging. 2008;27:1–7. 18050325 10.1002/jmri.21087

[33] Pagani E Agosta F Rocca MA. Voxel-based analysis derived from fractional anisotropy images of white matter volume changes with aging. Neuroimage. 2008;41:657–67. 18442927 10.1016/j.neuroimage.2008.03.021

[34] Bennett IJ Madden DJ Vaidya CJ. Age-related differences in multiple measures of white matter integrity: a diffusion tensor imaging study of healthy aging. Hum Brain Mapp. 2010;31:378–90. 19662658 10.1002/hbm.20872 PMC2826569

[35] Lazar M Alexander A Thottakara P. White matter reorganization after surgical resection of brain tumors and vascular malformations. Am J Neuroradiol. 2006;27:1258–1271. 16775277 PMC8133916

[36] Brozoski TJ Brown RM Rosvold H. Cognitive deficit caused by regional depletion of dopamine in prefrontal cortex of rhesus monkey. Science. 1979;205:929–932. 112679 10.1126/science.112679

[37] Cohen JD Perlstein WM Braver TS. Temporal dynamics of brain activation during a working memory task. Nature. 1997;386:604–8. 9121583 10.1038/386604a0

[38] Murphy B Arnsten A Goldman-Rakic P. Increased dopamine turnover in the prefrontal cortex impairs spatial working memory performance in rats and monkeys. Proc Natl Acad Sci. 1996;93:1325–1329. 8577763 10.1073/pnas.93.3.1325 PMC40079

[39] Owen AM, The functional organization of working memory processes within human lateral frontal cortex: the contribution of functional neuroimaging. Eur J Neurosci. 1997;9:1329–39. 9240390 10.1111/j.1460-9568.1997.tb01487.x

[40] Sawaguchi T Goldman-Rakic PS. D1 dopamine receptors in prefrontal cortex: involvement in working memory. Science. 1991;251:947–50. 1825731 10.1126/science.1825731

[41] Simon H. Dopaminergic A10 neurons and frontal system (author’s transl). J Physiol. 1981;77:81–95. 6785426

[42] Zahrt J Taylor JR Mathew RG. Supranormal stimulation of D1 dopamine receptors in the rodent prefrontal cortex impairs spatial working memory performance. J Neurosci. 1997;17:8528–35. 9334425 10.1523/JNEUROSCI.17-21-08528.1997 PMC6573725

[43] Bäckman L Nyberg L Lindenberger U. The correlative triad among aging, dopamine, and cognition: current status and future prospects. Neurosci Biobehav Rev. 2006;30:791–807. 16901542 10.1016/j.neubiorev.2006.06.005

[44] Baarendse PJ Counotte DS O’donnell P. Early social experience is critical for the development of cognitive control and dopamine modulation of prefrontal cortex function. Neuropsychopharmacology. 2013;38:1485–94. 23403694 10.1038/npp.2013.47 PMC3682143

[45] Van Kerkhof LW Damsteegt R Trezza V. Social play behavior in adolescent rats is mediated by functional activity in medial prefrontal cortex and striatum. Neuropsychopharmacology. 2013;38:1899–909. 23568326 10.1038/npp.2013.83 PMC3746695

[46] Machanda ZP Rosati AG. Shifting sociality during primate ageing. Philos Trans R Soc London Ser B. 2020;375:20190620. 32951557 10.1098/rstb.2019.0620 PMC7540961

